# Institutional-level drivers of gender-inequitable scientific career progression in sub-Saharan Africa

**DOI:** 10.1186/s12961-021-00767-1

**Published:** 2021-08-17

**Authors:** Millicent L. Liani, Isaac K. Nyamongo, Justin Pulford, Rachel Tolhurst

**Affiliations:** 1 Department of International Public Health, Liverpool School of Tropical Medicine, Pembroke Place, Liverpool, L35QA United Kingdom; 2 Faculty of Health and Life Sciences, University of Liverpool, Liverpool, L69 3BX United Kingdom; 3 Division of Research and Innovation, The Cooperative University of Kenya, Karen, P.O. BOX 24814-00502, Nairobi, Kenya

**Keywords:** Researchers’ lived experiences, Institutional environment, Macro-level factors, Gender equity, Intersectional gender analysis, Social power relations, Scientific career progression, Sub-Saharan Africa, Health research capacity-strengthening

## Abstract

**Background:**

This study sought to determine how institutional environments, including values, policies, and their implementation, shape inequities in scientific career progression for women and men, and their disadvantages in relation to their multiple social identities in sub-Saharan Africa (SSA). The findings are drawn from a wider research study that was aimed at gaining an in-depth understanding of the barriers and enablers of gender-equitable scientific career progression for researchers in SSA. This was nested within the context of the Developing Excellence in Leadership, Training and Science in Africa (DELTAS Africa) programme—a health-based scientific research capacity-strengthening initiative.

**Methods:**

The study adopted an exploratory qualitative cross-sectional study design. In-depth interviews (IDIs) with trainees/research fellows at various career stages supported and/or affiliated to three purposively selected DELTAS Africa Research Consortia were the main method of data collection. In addition, key informant interviews (KIIs) with consortia research leaders/directors, co-investigators, and the consortia management team were also conducted to corroborate information gathered from the IDIs, and also to provide additional insights on the drivers of intersectional gender-inequitable career progression. In total, 58 IDIs (32 female and 26 male) and 20 KIIs (4 female and 16 male) were conducted. The interviews were carried out in English between May and December 2018. The data were analysed inductively based on emergent themes.

**Results:**

Three interrelated themes were identified: first, characterization of the institutional environment as highly complex and competitive with regard to advancement opportunities and funding structure; second, inequitable access to support systems within institutions; third, informal rules—everyday experiences of negative practices and culture at the workplace, characterized by negative stereotypical attitudes, gender biases, sexual harassment, and bullying and intimidation.

**Conclusions:**

We contend that understanding and addressing the social power relations at the meso-institutional environment and macro-level contexts could benefit career progression of both female and male researchers by improving work culture and practices, resource allocation, and better rules and policies, thus fostering positive avenues for systemic and structural policy changes.

## Background

Health research capacity-strengthening (HRCS) initiatives have been identified as critical drivers for creating a large number of well-trained health researchers and institutions in low- and middle-income countries (LMICs), including sub-Saharan Africa (SSA) [1]. These efforts have seen substantial investments from various donor agencies [2], with a shift in focus from international to local leadership of training programmes in SSA [3]. A key mandate for many of these international HRCS programmes has been to develop and facilitate academic scientific research career pathways, with the anticipation that the established local investigators will train and mentor future cadres of investigators and research leaders [3]. Indeed, recent developments by funding bodies have led to a renewed interest in understanding the gender equity concerns in career progression of fellowship recipients and their retention in academic scientific career paths [4, 5]. Despite the existence of several HRCS programmes in SSA, we have not come across a study that provides in-depth explanations on the existence of such concerns along the scientific career pathways for researchers who are beneficiaries of such programmes within their institutions. A promising research capacity-strengthening initiative requires a gender equity lens, since compared to men, female researchers are often disadvantaged in pursuing scientific research careers and accessing senior leadership positions [6, 7].

Scholars have argued that gendered power relations affect women’s everyday experiences once they enter the academic scientific workforce; they may be subjected to sexual harassment, exclusion from career development opportunities, prejudices concerning their academic abilities and intellectual authority, and unconscious biases among others [8]. Therefore, to inform action for institutional change, it is important to gain insights into their experiences to understand the underlying institutional-level drivers and processes that produce gender inequities in science careers in the context of African academic and scientific research institutions [9]. In doing so, there is an increasing recognition of the need to go beyond the binary notion of gender, towards embracing an intersectional approach to gender analysis, which is critical to understanding the way different social strata and power structures produce inequities in career progression for both female and male research scientists [9].

It is against this backdrop that we sought to explore the institutional-level drivers of gender-inequitable scientific career progression as experienced by female and male researchers, and their disadvantages in relation to their multiple social identities in SSA. The data presented are part of a wider qualitative research study set within the context of the Developing Excellence in Leadership, Training and Science in Africa (DELTAS Africa) programme, an HRCS initiative. The details of this 5-year (2015–2020) programme were presented in another paper [10].

## Theoretical and conceptual framing

**Fig. 1:**
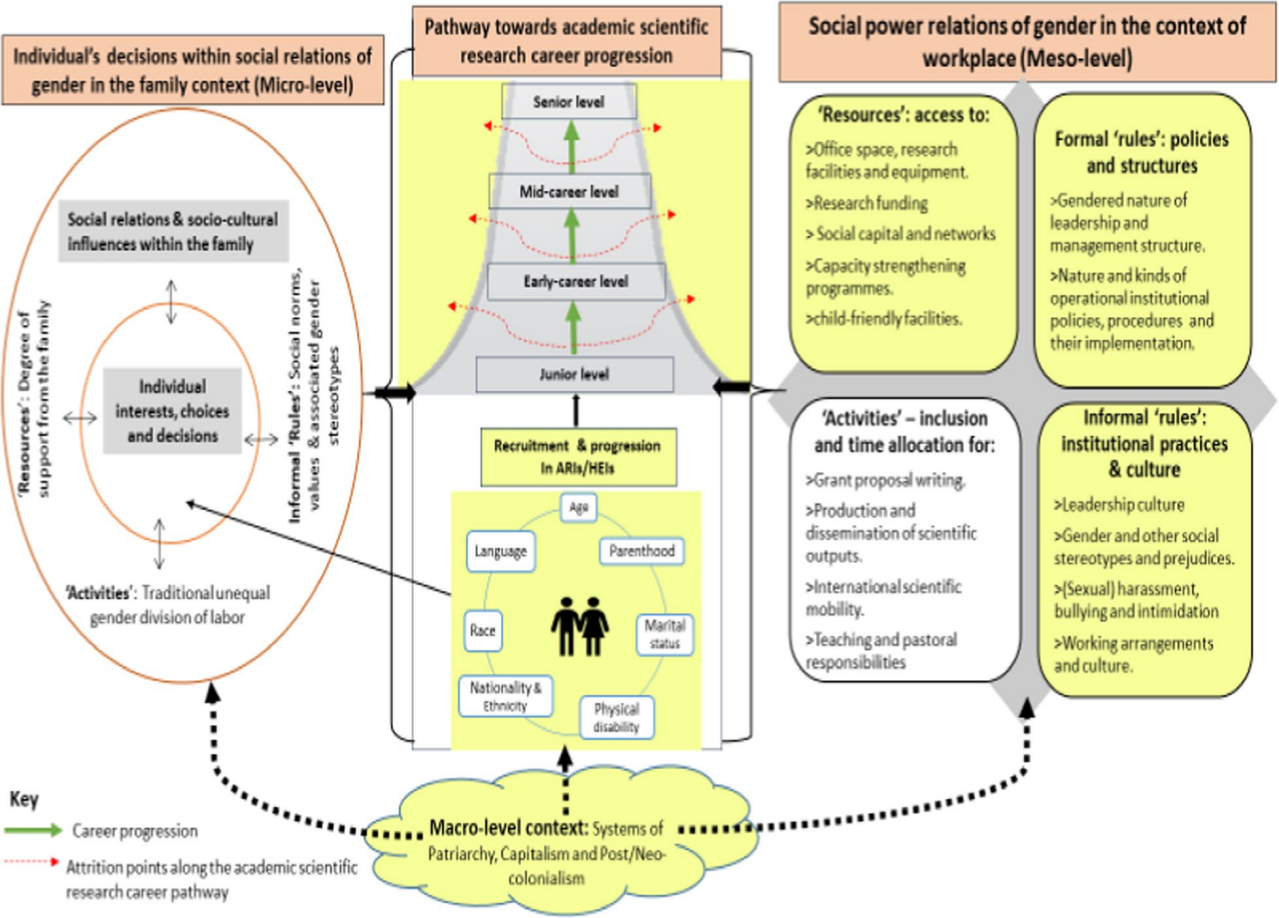
An integrated conceptual framework for understanding intersecting gender inequities in academic scientific career progression in higher education institutions (HEIs) in SSA. *ARIs* African research institutions

The empirical research for this study was informed by three theories and models: Systems of Career Influences Model [11], the Social Relations Approach [12–14], and intersectionality theory [15, 16] (see also [9]). These three theoretical and conceptual models were drawn together to form an integrated conceptual framework [9] which was developed based on existing evidence around the current research problem within the context of SSA as presented in Fig. 1 below.

The Systems of Career Influences Model [11] provides the central core of the framework, which focuses on the interplay between sociocultural influences within the family and organizational factors in shaping career advancement among women at different career stages. Kabeer’s framework on the Social Relations Approach [12] provides key dimensions for an institutional gender analysis—within the family and workplace, expressed as “rules” (formal and informal), “resources”, and “activities”, which are all permeated by “power”. “People” are located as individuals at the centre of the family and as entrants into the career pathway. The intersectionality lens [15, 16] is then explicitly added to highlight the multiple social identities and related power of these individuals according to aspects such as age, professional cadre, marital status, ethnicity/race, and parenthood.

We used this integrated conceptual framework as a lens through which to understand the everyday experiences of individual researchers who are characterized by multiple social identities with their science careers as they relate to institutional environment, policies, and practices, as well as access to the necessary research infrastructure or “resources” [9]. We have taken gender as a key entry point into analysing the positionality and experiences of individual researchers, who according to an intersectionality perspective may be further identified as (dis)advantaged based on other multiple intersecting social categories. Such individuals may either see their careers stagnate or decide to opt out of the scientific career path. Specifically, as indicated in the components of the framework highlighted in yellow, we focus on how social power relations of gender in the context of the workplace—meso-level (right box)—exacerbated by macro-level systems of power (word bubble) shape the everyday experiences of female and male scientific researchers in SSA characterized by multiple social identities (middle box) to progress along the academic scientific career ladder.

## Methods

### Study design and setting

An exploratory qualitative cross-sectional study design was adopted. The research was conducted within the context of the DELTAS Africa initiative. The programme is coordinated by the African Academy of Sciences’ Alliance for Accelerating Excellence in Science in Africa (AESA) 1 and implemented by a network of 11 African-led health research programmes, commonly referred to as DELTAS Africa Research Consortia (DELTAS ARC). The DELTAS ARC offers collaborative research training programmes in various scientific disciplines, ranging from biomedical to social sciences, spanning 54 lead and partner institutions (research organizations and universities) across SSA, in partnership with Northern academic institutions. In doing so, it facilitates career development of postgraduate science students (masters and doctorate), both of whom are referred to in this study as junior researchers, and scientific research professionals (postdoctoral fellows and mid-level researchers), who pursue research work/studies at institutions in their home—or other African—countries.

This study adopted a two-tiered purposive sampling strategy for selection of consortia and participants within the sampled consortia. This was based on the principles of maximum variation sampling, which allowed us to discover patterns for core elements or dimensions that hold across our diverse sample, as well as unique or distinctive variations [17]. The first step involved purposive sampling of three DELTAS ARC. These were selected based on the following: regional representation in SSA (Eastern Africa, Southern Africa, and West and Central Africa); representation of consortia that are located in English- and French-speaking countries; presence of fellows of diverse nationalities recruited from different African countries; and consortia with the presence of fellows at various career stages, from masters (Msc), doctoral (PhD), and postdoctoral research fellows (PDF) to mid-career research (MCR) scientists.

In each of the purposively sampled DELTAS ARC, we sought heterogeneity by using gender as a primary selection criterion for in-depth interview (IDI) study participants. Other multiple social identities were sought along axes of career stage, scientific discipline, duration in the programme/institution, and nationality. A list containing such information was provided by the research directors of the sampled DELTAS ARC, which aided in purposive selection of study participants. We collected additional information about personal identities including age, marital status, and presence of children by administering a brief questionnaire before commencement of IDIs. During the interviews, we asked the participants to reflect on how such identities shaped their everyday experiences of science careers with respect to institutional environment, policies, and practices. Key informants were selected based on their role and knowledge about the functioning and operation of their respective DELTAS ARC.

### Data collection methods

The IDIs with trainees/research fellows at various career stages supported and/or affiliated to the DELTAS ARC were the main method of data collection. This was aimed at exploring qualitative narratives about everyday lived experiences of how institutional environments, including values, policies, and their implementation, shape inequities in scientific career progression for female and male researchers in SSA characterized by multiple social identities. IDIs mainly focused on the personal experiences, perspectives, and understanding of individuals with regard to their career progression as DELTAS-funded fellows and took a narrative approach to allow gendered dimensions to emerge from the participants’ stories [18]. Key informant interviews (KIIs) with consortia research leaders/directors, programme managers/coordinators, monitoring and evaluation officers, and supervisors (co-investigators) were also conducted. This was designed to corroborate information from the IDIs and to provide additional information on the drivers of intersectional gender-inequitable career progression within the wider institutional environment. In line with Patton’s characterization of key informants [17], we selected persons who were particularly knowledgeable about the wider study setting from the different vantage points of management, and conducted semi-structured interviews with these informants to explore specific issues in the selected institutions. In total, 58 IDIs (32 female and 26 male) and 20 KIIs (4 female and 16 male) were conducted across the three purposively selected DELTAS ARC. Most IDIs (*n* = 47/58) and KIIs (15/20) were conducted in person by the lead author (ML), a social science doctoral candidate with extensive experience in conducting interviews in qualitative research, at the respective consortia secretariat or annual scientific meeting. The remainder were conducted via Skype or telephone. The interviews were conducted between May and December 2018, all in English. Despite making provisions for a bilingual research assistant who was fluent in writing and speaking English and French to help in conducting some interviews in French, all the Francophone study participants expressed that they were comfortable conversing in English as opposed to using a translator. Data saturation was attained after conducting 58 IDIs and 20 KIIs with eligible participants, as no new information was being obtained. All interviews were audio-recorded using a digital voice recording device, alongside note-taking. On average, the IDIs lasted 90 minutes, while KIIs took 75 minutes.

### Characteristics of the IDI sample

The IDI study participants were nationals of 13 SSA countries across Eastern (Uganda, Kenya, Rwanda, Somalia), Southern (Zambia, Botswana, and South Africa), and West and Central Africa (Senegal, Ghana, Nigeria, Benin, Mali, and Cameroon). They represented three consortia comprising 11 partnering institutions, of which seven were research institutes and four were African public universities. The majority identified English as their everyday language of scientific communication (52/58), while the rest reported French. Regardless of gender, only a few participants (9/58), particularly at the PDF and MCR levels, held faculty positions, mainly as lecturers and assistant professors. Overall, the majority of study participants identified as biomedical scientists (45/58), while the rest were social scientists (13/58). Regardless of gender, most study participants were from less educated family backgrounds (46/58), where no parents or siblings had attended university. More female than male participants had young children, and women at the early career stages were more likely than men to have young children. Table 1 summarizes the general sociodemographic characteristics of the IDI study participants.

**Table 1:** Sociodemographic characteristics of the IDI study participants (*n* = 58)

Gender	Other characteristics	Total (*n* = 58)	MSc (*n* = 14)	PhD (*n* = 19)	PDF (*n* = 18)	MCR (*n* = 7)
Women (*n* = 32)						
Age range	25–29	9	7	2	–	–
	30–34	12	2	9	1	–
	35–39	5	–	–	2	3
	40–44	4	–	1	2	1
	45–49	2	–	–	1	1
	Total	32	9	12	6	5
Marital status	Unmarried^a^	16	7	4	3	2
	Married	16	2	8	3	3
	Total	32	9	12	6	5
With children < 5 years	Unmarried (16)	4/16	0/7	0/4	2/3	2/2
	Married (16)	12/16	2/2	6/8	3/3	1/3
	Total (32)	16/32	2/9	6/12	5/6	3/5
Family educational background^b^	Highly educated	8	2	2	1	3
	Less educated	24	7	10	5	2
	Total	32	9	12	6	5
Men (*n* = 26)						
Age range	25–29	4	3	1	–	–
	30–34	8	2	3	3	–
	35–39	9	–	3	5	1
	40–44	2	–	–	2	–
	45–49	3	–	–	2	1
	Total	26	5	7	12	2
Marital status	Unmarried^a^	11	5	4	1	1
	Married	15	–	3	11	1
	Total	26	5	7	12	2
With children < 5 years	Unmarried (11)	0/11	0/5	0/4	0/1	0/1
	Married (15)	11/15	0	1/3	10/11	0/1
	Total (26)	11/26	0/5	1/7	10/12	0/2
Family educational background^b^	Highly educated	4	1	1	2	0
	Less educated	22	4	6	10	2
	Total	26	5	7	12	2

^a^The label “unmarried” includes those who identified themselves as single (never married), divorced, or separated. We grouped them together in order to protect participants’ anonymity and confidentiality, particularly for the latter two identities

^b^We based this on the parents’ and siblings’ level of education, with those who had attended university considered as highly educated

#### Data processing and analysis

All audio data were transcribed verbatim by an experienced qualitative research assistant. The transcripts were verified by comparing the audio files and scripts with the field notes. Once this process was complete, transcripts were sent to all individual study participants for member-checking to ensure participants’ views were appropriately captured. This process also allowed the participants to identify content they preferred to be removed from the analysis, such as individual characteristics and statements that they felt might easily identify them. Following the member-checking process, most of the IDI participants asked to have the identities of their ARC and affiliated institution, number of children, country of origin, and disciplinary field of study withheld for confidentiality purposes. In addition, they suggested that findings be presented as views and experiences of participating DELTAS Africa research fellows as a whole. In protecting participants’ anonymity and confidentiality, all identifiers have been replaced with pseudonyms. However, given the necessity of an intersectional gender analysis, other identities such as age (provided in range), marital status, and presence of dependents are anonymously presented where necessary. Thereafter, the data were organized and coded in QSR International’s NVivo 11 qualitative data management software, and analysed inductively based on emergent themes and the relationships between them as presented in a conceptual framework reflected in the results and discussion section. We utilized a grounded theory approach, employing constant comparative analysis [19, 20]. All illustrative quotes have been carefully reviewed for their potential to reveal individual identities.

## Results

Three interrelated themes were identified. They illustrate how women’s and men’s everyday lived experiences within their workplace environment are shaped by institutional power relations underpinned by macro-level forces of patriarchy, capitalism, and neocolonialism. This leads to a highly complex and competitive environment characterized by limited access to the necessary research resources, and dissatisfaction with operational policies and power structures (formal rules) as well as institutional practices and culture (informal rules). In this process, gender intersects with other aspects of identity, leading to differing work experiences and inequities in career progression.

### Theme 1: Complex and competitive institutional environment: Progression opportunities and funding structure

The participants’ narratives about their everyday experiences within the institutional environment revealed how global and national political economies, shaped by neocolonialism, influence institutional funding models. At the funding national and institutional levels, positional hierarchies within institutions are reinforced by racism, ageism, nepotism, and patriarchy, shaping the way the funding environment is experienced. All these axes of inequity intersect with institutional policies, practices, and culture, creating a highly complex, competitive, and insecure work environment characterized by limited career progression opportunities and uncertainties with research funding. Consequently, scientific research was consistently perceived by most female and male participants at all career stages as a “very scary career” characterized by short-term research contracts, resulting in job insecurity and financial instability. This further exacerbates inequities at the micro level of family, creating difficulties for women and men in fulfilling their normative gender roles, with differential implications and outcomes for researchers’ career progression and personal well-being, as presented in a different paper [10].

#### Uncertainties with research funding and the resultant implications

Most female and male participants at all career stages stated that research funding was essential for scientific career progression. Some participants, particularly at the early and mid-level career stage, attributed the research funding uncertainty they experienced to racial inequities in international grant allocation and stiff competition. They perceived racial discrimination by international funding agencies in grant funding for African applicants, commenting that it is hard to win a research grant as a lone African applicant without a White collaborator/co-applicant:

*The fellowships are very competitive…most African researchers and applicants here feel like we actually don’t get funded because we are Africans…there is always some barrier towards being awarded a fellowship or a grant if you are only African applicants. But at least if there is a European or White co-applicant, then the application seems to be successful. (IDI, Female, #28, MCR)*

In the same vein, another participant noted that on several occasions he had heard his colleagues say, “If your supervisors are all Blacks, then you wouldn’t easily progress in your career because there is a notion that even if we apply, we are unlikely to get the funding” (IDI, Male, #22, PDF). Such concerns were corroborated by a key informant who reported, “Most senior fellowships are still skewed towards whites…this is an issue that I have observed for close to 15 years I’ve been here (research institution) … for whatever reason (funding agencies) say that they have difficulties attracting African fellows” (KII, Male, #08).

Participants also expressed concerns about the merit criteria for application for research funding, which are sometimes conditioned on holding a faculty position, which they felt places many African researchers at a disadvantage. Notably, most participants in this study were affiliated to research institutions, the majority of whom did not have faculty positions. For example:

*Here in Africa, most research scientists like us who work in research institutes do not hold faculty positions in universities…so if the funder puts a condition where you need to be a faculty staff somewhere as part of the requirement for a grant application, sometimes this discourages you from applying…you feel you are not good enough…they already have the condition that disqualifies you from applying, or even if you apply, you are likely to be unsuccessful. (IDI, Male, #11, PDF)*

Consequently, the competitive nature of the grant application and allocation process, coupled with scarcity of financial resources, created anxiety for most participants about continuing on this career path. Indeed, most researchers at different career stages, whether female or male, and irrespective of their marital or parental status, expressed concerns about the likelihood of ongoing short-term employment contracts and few prospects for permanent appointments. This creates job insecurity and financial instability, making progression in scientific research career path unappealing:

*It’s a very scary career…You are always thinking about if this contract runs out, where am I going to next? Will I go to another research institution? Will I get another research project that I will work on? …that uncertainty and the fact that I am a married man with a family to feed and you are always given short-term contracts with no job security is something that can really distract you from staying in this career path. (IDI, Male, #23, PDF, married, under-5-year-old children)*

However, these common fears had clear gendered dimensions. For example, anxiety about financial insecurity was considered by most male participants as their most pressing challenge, particularly given their societal expectations to fulfil the breadwinning responsibilities for their families. Notably, most of them identified themselves as coming from low socioeconomic family backgrounds, whose extended family members were financially dependent on them for support with living costs, as well as paying school fees for their younger siblings. Moreover, female participants who were single parents with no additional family income also perceived this as extremely challenging:

*For me, the main challenge has mainly been financial impact. I don’t think I will be writing grants for the rest of my life because the possibility of sailing through is slim…that is not the direction I want to go with my science. This is all complicated…for a single mother like myself, you must figure out how your child will survive…perhaps if I was married, it would be easier as you would have complementary family income… I am contemplating to move into entrepreneur or in an NGO [nongovernmental organization] that implements projects, if things get tough. (IDI, Female, #25, PDF, unmarried, under-5-year-old child)*

Overall, in his reflections about this problem of funding uncertainty and job insecurity, a junior research fellow highlighted that his mentor always advises him that “science is not for the faint-hearted…if you are looking for financial stability, then you shouldn’t be in sciences” (IDI, Male, #09, PhD).

#### The “hustle” for career progression opportunities

Related to uncertainty with research funding were concerns about the limited career progression opportunities in science in Africa, which most female and male participants felt was a “hustle” due to its highly competitive culture. From the perspective of career progression within research institutes, a participant noted, “If you don’t have a grant, you can’t be guaranteed a working space in the lab within a research institution” (IDI, Male, #14, MCR). In addition, most participants were also concerned about the dearth of strong research institutes in Africa, which makes it difficult to enhance career progression for all trained fellows. This problem was also attributed to the limited investment in research by most African governments, indicating how macro-level political economic forces at the national level shape inequities, which may limit researchers’ progression in such a career path.

*The government in many African countries aren’t ready to invest into research… So that again for me is a limitation. If you want to continue in this career path, especially on this continent, how feasible is that going to be? If we are not going to have access to funding like the one we are currently having through DELTAS, it is difficult to envision career progression based in what actually happens in our African context. (IDI, Male, #08, PhD)*

The lack of core funding for research by the African governments culminates in a dearth of advancement opportunities, resulting in an undefined research career pathway, particularly in African universities, as most faculty members focus primarily on teaching while they conduct research “on the side”. Indeed, through their detailed description of the situation in most SSA universities, most participants attributed the dearth of opportunities for early-career researchers to transition into faculty positions to the lack of a structured approach to career progression and succession planning. This results in ad hoc recruitment processes and limited and infrequent vacancies at some departments. The fact that most African universities rarely recruit junior faculty staff makes progression difficult for early-career researchers who desire to take an academic scientific career path, but are left “hustling”, as illustrated by the quote below:

*In our department, the last time they recruited for junior staff was in 1990s. It was in 2017 that they had one vacancy for professor of entomology… I would like to have a position in the university, but it is not easy to have junior faculty positions advertised…This is a very big problem here and in Africa in general…If you finish your PhD and postdoctoral fellowship, you don’t have somewhere to go…you keep hustling! There are no opportunities. (IDI, Female, #04, PDF)*

This problem was shaped and reinforced by other axes of power such as tribalism, nepotism, and an aging workforce remaining in posts. For example, most participants observed that tribalism and nepotism were commonly practiced by senior university management staff, who promote the recruitment of their relatives and those with whom they are ethnically affiliated for junior faculty positions. On the other hand, discrimination based on age revolved around professors holding on to their positions despite their advanced age, which was reinforced by institutional policies that do not enforce the retirement age, hindering the entry of junior researchers into the academic career pipeline. As one participant noted, “It is not easy to get an academic position unless a professor dies” (IDI, Male, #13, MCR). The pervasiveness of age-related hierarchies and reluctance to breach these on merit criteria perpetuated further inequities in providing faculty positions for junior and early-career researchers. Such experiences and observations were alluded to by a key informant who noted that most African universities operate on an “old deadwood” model of lifetime positions for professors. This creates difficulties in hiring young, ambitious academic scientists to drive the research field forward, as illustrated in the following quote:

*Universities in Africa operate on an “old deadwood” model where once you are in your job, you never leave. You can just stay in it forever irrespective of how effective you are…there is no oversight on how to ensure quality and rigorous progressive science…Therefore, some institutions stagnate to hire emerging academics because there is just all this old deadwood, with no space or money for young scientists who are ambitious to drive science forward. (KII, Male, #14)*

The lack of career guidance on possible career pathways within and outside academic scientific research by the DELTAS Africa initiative, as reported by most female and male doctoral research fellows, compounds this issue. One participant noted, “There are ‘a lot of hanging things on next steps’…it is not clear what the path is for us when we finish…there is no career advice on where to go next… you are left to plan on your own” (IDI, Male, #20, PhD). To them, career progression to the next level “could be dependent on how merciful your supervisor would be by offering you a position, one you finish” (IDI, Female, #14, PhD). Another participant further stated:

*I don't think people [research leaders] have spent time sitting down to offer us communication and career advice on what do you do next after PhD completion… It would be nice for someone to come and talk to you about what are the various career options. (IDI, Female, #20, PhD)*

Consequently, as was noted, “If there is no clear pathway in science for fellows, it is likely for them to move elsewhere [out of science]” (IDI, Male, #11, PDF). Moreover, the risk of brain drain for excellent trained African scientists to the Global North is likely to occur, as illustrated by the following quote:

*We don’t have a good career path here in Africa…there is no tenure, there is no job for life, there is no pension, you are just entirely hustling. And so, if they go to the Europeans or Americans, they will give you tenure, lectureship or something better which is much more attractive…then they begin to see a clear trajectory which doesn’t maybe exist in their home countries... that is a massive problem for retention of African research scientists. (KII, Male, #19)*

### Theme 2: Inequitable access to support systems within institutions

This theme elucidates participants’ narratives on the ways that social power relations of gender shaped their everyday lived experiences at the workplace in accessing relevant resources and how informal rules of institutional practices and culture exacerbated inequities in career progression.

#### Insufficient social resources: mentoring and a dearth of female role models

One of the mandates of the DELTAS Africa initiative is to provide mentorship to research fellows as a mechanism for enhancing career progression in science, which most participants underscored as “crucial during the early career stages” (IDI, Male, #06, MSc). Important roles of mentors were described as providing advice on how to progress in one’s career, keeping an eye on mentees’ social well-being, and supporting with linkages to the right professional networks and research collaborators. However, some female and male participants perceived that they had received insufficient mentoring across the science career trajectory. This was a common and multifaceted problem experienced by both women and men.

A common generic issue that emerged, particularly from two study sites/consortia, was that not all fellows were assigned mentors. This was partly due to a lack of structured mentorship programmes in place to facilitate the process. Where mentors were assigned, some mentees noted that they rarely met with them, not even virtually, since the mentors were extremely busy. The affected participants complained that they were allocated mentors without involvement in their selection, leading to a mismatch due to personality or other differences and a consequent lack of one-on-one relationships.

From a gender perspective, some female participants reported that it was commonly assumed that supervisors and thesis advisory committee members, the majority of whom were male, could also simultaneously serve as “natural” or “automatic” (IDI, Female, #03, PhD) mentors. This presented additional problems, since when faced with personal (e.g. failed and broken relationships, difficulties with work/life balance) and work-related problems (e.g. abusive supervision, sexual harassment) that affected their careers, they did not feel comfortable sharing these with male mentors. Some expressed the need for female mentors to provide psychosocial support, emphasizing that “sometimes you need to have someone who understands you, who is married and easy to relate with” (IDI, Female, #14, PhD, married, under-5-year-old children). In particular, most junior and early-career female research fellows without children frequently expressed the need for support and guidance on how to manage the common dilemma expressed by female researchers about how to progress in science alongside the anticipated pressures of childbearing and child-rearing responsibilities for women. For example: “What if I get pregnant! How will I progress in science?” (IDI, Female, #22, PhD, unmarried). Some women with children also expressed the need for mentorship on managing critical career transition points whilst they had dual responsibilities for young children. For example:

*Transitioning from postdoctoral research fellowship to a principal investigator is very difficult…to me, this is a career stage where you need someone to genuinely encourage you, mentor you, give you the right kind of support on how to deal with family and research career. (IDI, Female, #27, PDF, married, under-5-year-old children)*

Overall, most key informants stated that they encouraged both female and male fellows to find informal mentors for themselves who could support them with career progression, although most women had an additional challenge finding female mentors because of the smaller number of senior female research scientists.

Role models were also recognized as important to aspiration and strategic direction in research careers. In this study, most male participants considered either their male supervisors, mentors, or the DELTAS consortium leaders as their role models in scientific careers. In contrast, most female researchers, especially at the junior and early career stage, expressed that they rarely had female role models in science. Instead, they commonly pointed to their DELTAS consortium research leaders, and to some extent supervisors and mentors, most of whom were male. When prompted to reflect on the lack of female role models amongst those they identified, they frequently noted that they had very few examples of women in senior scientific positions who were also in successful marriages, since it seemed that most of them had to sacrifice their marriages to enable them to advance in their careers:

*But I don’t see any successful, powerful and huge women as science directors that are still in their marriage and who have maintained a successful family life! ...It seems someone has to sacrifice something! Something must fall apart one way or the other…realistically speaking, I think my family would definitely suffer if I became more ambitious in science… for me that would be the hindrance, I would say. (IDI, Female, #11, PhD, married, under-5-year-old child)*

Many female junior and early-career researchers who were already married or were planning to get married and establish families said that such observations led them to doubt whether they could follow careers in scientific research. Growing up within an African patriarchal context, with a strong linkage between marriage and childbearing responsibilities for women, those who had sacrificed such expectations for their career progression were labelled by some female researchers as poor role models or mentors.

#### Inflexibility of formal rules around work policies and culture

There was consensus amongst key informants that formal flexible work policies within their institution do not exist, although research fellows were perceived as “usually” able to make informal arrangements with their supervisors on provision of flexi-time. However, some informants admitted that:

*The culture and practice of flexible working arrangement is more for the senior level researchers, from postdoctoral research fellows moving upwards…this can be extremely difficult for postgraduate research fellows. (KII, Male, #05)*

A common issue raised by most female and male research fellows at all career stages was their dissatisfaction with the way in which flexible work opportunities may depend on one’s position within the institutional hierarchy. This was acknowledged by both women and men as a particularly acute problem for women:

*The reality is that flexibility mainly depends on the level at which one is located at the science professional cadre. A woman who is not in senior position wouldn’t be comfortable to keep requesting the supervisor for flexi-time, as not all supervisors are the same at granting such opportunities. (IDI, Female, #31, MCR)*

Considering that women bear the brunt of reproductive responsibilities in their everyday lives compared to men, the lack of formal provision of flexible working hour policy or procedure within the institutions was seen by some women as “gender-discriminatory issues …through unconscious biases from the leadership with no conscious considerations on how it could impact on career pathways” (IDI, Female, #05, PDF, married, under-5-year-old children). A male participant also noted that “keeping women in science careers, and who have reproductive duties to fulfil without provision of formal flexible working arrangement, is just a dream” (IDI, Male, #12, PDF, married, under 5-year-old children). He further placed emphasis on this issue as particularly challenging for women, asserting that provision of flexible work opportunities by institutions would also enable men to take on and assist women with reproductive and care responsibilities.

#### Lack of institutional support for female researchers with nursing needs

The absence of mother- and baby-friendly lactation rooms at the workplace presented difficulties for female researchers with nursing needs, which they expressed as indicative of the gender insensitivity of the workplace environment. Some female participants with young children lamented, “If you don’t have a personal office or a car and happen to be a nursing mother, it is hard to find a conducive place to express and store breast milk while at work” (IDI, Female, #25, PDF). When caught in such a situation, a common option for them was to use bathrooms for breast milk expression as well as storing the milk in a common refrigerator, which to them was unhygienic. Even where an individual manager was sympathetic, the physical environment was unconducive. In one consortium, a male supervisor (interviewed as a key informant) explained that he improvised by allowing his supervisee to use his office for nursing, but since his office was glass-walled, he had to cover the walls with papers to enable privacy. Overall, this finding was corroborated by most key informants, who admitted that provision of adequate and well-equipped lactation rooms within their institutions was lacking.

### Theme 3: Informal rules: Everyday experiences of negative practices and culture at the workplace

Some female participants across all career stages in the sampled consortia narrated their experience of an uncomfortable workplace environment characterized by negative stereotypical attitudes, gender biases, sexual harassment, and bullying and intimidation. They felt that this environment impeded their career progression within the institution, and/or could even lead to attrition in the scientific career sector. Such concerns were rarely experienced by the male participants, most of whom acknowledged that such issues were mainly experienced by women.

#### Negative stereotypical attitudes at work towards “career women” and social scientists

This was an issue that was mainly raised and experienced by some junior and early-career female researchers. For example, a participant complained about some female and male colleagues at her workplace who occasionally questioned her as to why she is still unmarried, implying that she was prioritizing her career over marriage, which made her feel uncomfortable:

*It is more individual colleagues who will make the workplace sometimes uncomfortable because they think at your age you should be married, you should have children…so sometimes, you know, they won’t say it directly but the message that is coming across is like you are prioritizing your career over other things. (IDI, Female, #27, PDF, unmarried)*

Sometimes formal meetings by female networks at the workplace were negatively perceived by male colleagues as gossip time:

*When we are having meetings with my friends at work, they [male colleagues] would think that all that we do as women is to gossip. And then when we start winning project proposals, then they are like, “You people, when you have projects and proposals, you only invite your friends!” My friends are all ladies. And when we are winning proposals, they are like, “you just gossip!” (IDI, Female, #06, PDF)*

Gendered disciplinary stereotyping for social scientists: Most participants who identified as social scientists perceived themselves as underappreciated minorities within their respective (largely biomedical research-focused) institutions:

*There isn’t really an appreciation by biomedical scientists of what social science brings to the table…it is still overlooked as of less interest in the science agenda… [But then] you get their request to help them have a paragraph in their proposal that needs some qualitative research work. They are like, “ooh, can you please write this paragraph for me?”…And when it is funded, its focus is to complement the other sciences… it is like an afterthought...the assisting part of research. It is like it can’t stand by itself. So that remains a big problem for us which keeps making me feel bad. (IDI, Female, #26, PDF, social scientist)*

Accounts of gender stereotyping of the disciplinary field of social sciences, which was viewed as mainly dominated by women, were also prominent. For example, a female social scientist admitted that she had occasionally heard sentiments conflating social science methodologies and female gossip, such as “*what have you women been discussing, and not what have you social scientists been discussing*” (IDI, Female, #26, PDF, social scientist).

#### “Hot” and “hidden”: Gender biases at the workplace

Existence of gender biases within the workplace, mainly against female scientists, was reported by some female and male participants. This was characterized as a “problem that is ‘hot’ [very common], ‘hidden’, entrenched within the system, and which is difficult to see and tell that it exists” (IDI, Male, #25, PDF). A range of manifestations were described, including preferential treatment by some principal investigators (PIs) towards hiring male researchers, and some male scientific managers cautioning female scientists to avoid pregnancy within the life cycle of a research project. For example:

*Sometimes you experience bad attitude of some managers because some will be like, they don’t encourage pregnancy. They are like, why are you getting pregnant, and you are a student? …They are male senior scientific staff. They are like you are supposed to be concentrating on your work, nothing else! So, when you get such comments, you are like okay, so I shouldn’t do this? I should put it on hold, finish, then I should go and do this other thing. (IDI, Female, #23, PhD, married, no children)*

In the same vein, an early-career male researcher asserted that he had observed gender discrimination in hiring where some male PIs exhibited unconscious gender bias against providing job opportunities to young female researchers even when they turned out to be the best candidates. He further noted that when having informal conversations with such PIs, they usually argued that “women are likely to go on maternity leave, which is useless to have them, even though they performed better at interviews” (IDI, Male, #02, PDF). Similarly, a female research scientist from a different DELTAS ARC noted, “I heard a comment where somebody [PI] said that ‘I prefer hiring research assistants that are male because they don’t have to deal with things like pregnancies’” (IDI, Female, #32, MCR). Such attitudes result in feelings of guilt among junior female researchers, with some perceiving a need to “pause” their science career to have children or focusing on teaching instead of research.

#### Sexual harassment, bullying, and intimidation

Sexual harassment: This was experienced by some female researchers mainly at the junior and early career stages, most of whom identified as unmarried. However, when encouraged to elaborate further, most of them highlighted that they were uncomfortable speaking about it while still in the fellowship programme. As one participant said, “Personally I have sexually been harassed on several occasions…I really don’t want to talk about it…maybe after I am done and out of this place” [clicks—indicating how unbearable this problem was for her] (IDI, Female, #21, PhD, unmarried). A few opened up to share their experiences of sexual harassment by some male senior research scientists within their institutions. This took the form of physical sexual advances and sexual coercion, where a career progression opportunity was offered conditional on sexual activity.

When asked about whether they had a chance to report it to the relevant authorities, most were unaware of any sexual harassment policy for the institution in which they were affiliated to, noting that they had never been given an induction or even a handbook with such information. Additionally, they highlighted the lack of clear institutional procedures for how to report and effectively address such issues at both the institutional and consortium levels. Consequently, the affected participants feared that reporting or even speaking out would jeopardize their prospects of career progression within the same institution and elsewhere given that most institutions are interlinked through research collaborations. Moreover, they also feared a lack of confidentiality in handling the matter, citing that they might later be victimized. For example:

*There is no proper approach on how to handle and report it… I don't want to go to the director that I am reporting my supervisor, that I wouldn't do! Unfortunately, we are human beings, and you will meet over a cup of coffee and someone whom you reported the matter to might mention that and perhaps my name mentioned too. If it gets to my supervisor again, it will make things worse …continuing to work and grow here or even in other related research institutions can become difficult…so I think that is my major point of concern. (IDI, Female, #17, MSc, unmarried)*

Whilst most key informants reported the existence of policies and reporting procedures on harassment and discrimination, most participants within the same institutions were unaware of them. When asked whether any incidence of sexual harassment had ever been reported by research fellows within their institutions, most key informants said there had never been such a case. However, one informant expressed the view, “In every institution where there are men and women, you will always get sexual harassment…it is all about ‘power’ and as a show of strength mostly coming from male lecturers who end up sexually harassing most of the female doctoral fellows that they supervise” (KII, Male, #20). He further emphasized that even where policies exist, fellows may be sceptical about the chances of them being implemented, due to the influence of social power relations which privilege perpetrators:

*Even though there is a disciplinary council with professors who sit on the panel, it is very difficult to dismiss a lecturer, as almost all cases end up being withdrawn…most perpetrators usually have political connections or inclinations with the [university and ministry of higher education] administration. (KII, Male, #20)*

Participants who experienced sexual harassment expressed how uncomfortable they felt in the institutions; some considered opting out of the fellowship programme, while others noted that they would not like to pursue any future career progression opportunities at their current institution or programme.

Bullying and intimidation: This was experienced by some female fellows at various career stages. They asserted that this was mainly perpetrated by both female and male supervisors and senior research scientists towards junior, early-career, and mid-level female scientists, suggesting that workplace hierarchies were the most significant power relation at play. Notably, the kind of bullying experienced was often “more subtle and silent, which is hard to report, as there is no dictionary definition to it” (IDI, Female, #03, PhD). For instance, a female mid-career researcher attributed her own experience of bullying by senior scientists feeling threatened by her rapid career progression into their areas of expertise. This is indicative of the highly competitive nature of scientific culture in which researchers are expected to “fight” for their place, against those above them in the hierarchy as well as their peers. She further reflected on how women are often socialized to be more oriented towards “cordial relationships” and therefore less prepared to fight, which may lead to them opting out:

*I felt bullied. The bullying is very subtle because it is very low.… If you are an upcoming scientist trying to break through to senior level, stepping into a research area similar to that of your senior scientists makes them uncomfortable… there is a tendency sometimes to be bullied… I am encountering it right now. … It is more of power imbalances … you have to think of the checks and balances here. Obviously, the junior scientist doesn’t want to offend this one here because of mentorship and all that. You want a cordial relationship… [but] it is a battle which I don’t want to fight here! I just can’t fight! ...You decide this is not for me… for a man, they can fight over such issues without caring…they will say what they will say. For most women like me, we are very careful about what we say, and that doesn’t work very well. Women don’t “fight” good. And so, when you are encountering a situation where you have to fight, most women would just rather abandon the idea or just quit. (IDI, Female, #29, MCR)*

She further observed that bullying behaviours by seniors are one of the reasons why early- and mid-career-level researchers opt out of the scientific research career path.

Other bullying and intimidatory behaviours included yelling at junior fellows in public spaces within the institutions, as mainly reported by female fellows, and making demeaning statements to fellows and asking for a higher number of publications from junior fellows than required, which delays their graduation. For instance, a participant stated:

*I am literally terrified every time I am going to talk to him. He is going to tell me I am so stupid or, if he doesn’t use the word “stupid”, he says, “you are superficial, you are not thinking deeply”… It is so frustrating. Most of the time after talking to him, I can’t tell if I have made progress or not… sometimes I sit and cry because it is too much. (IDI, Female, #02, other identifiers withheld)*

Such behaviours were perceived as drivers of poor mental health. For example, a junior female researcher described how she had suffered from depression in silence and for which she sought treatment on her own.

*Very demeaning statements were said to me [by the supervisor] … I was getting drained day by day psychologically and emotionally … I had certain constant headaches and the doctors diagnosed depression…For three months, I wasn’t myself! …I was always getting medication without their [programme management team] knowledge. From the time I was suffering, I was just trying hard still to fight it… It was very difficult. (IDI, Female, #01, other identifiers withheld)*

She further noted that even though the programme had earlier assigned her a female mentor, she never disclosed to her how she was suffering in silence, as she did not have a personal rapport with her. Overall, such experiences could demotivate them from aspiring to advance their careers within the same institution or even lead to attrition among fellows in the programme and the specific institutions. Notably, none of the male participants reported experiences of bullying and intimidation.

#### Implications of women’s underrepresentation in scientific leadership and decision-making

Most female participants noted that direct and indirect discrimination, sexual harassment, bullying, and gender stereotyping result in fewer women progressing to scientific leadership and decision-making positions. Consequently, the lack of women in such positions acts as a barrier to changing institutional cultures and formal and informal rules:

*So, within our African institutions, it’s quite clear that there is a big problem. You will find the major executive and leadership positions are mainly held by men… they are not that much sensitive on issues about gender equality…people feel uncomfortable to reach out to them. Perhaps if we change the leadership towards including women, maybe this problem [sexual harassment] will be minimal. (IDI, Female, #32, MCR)*

In addition, a male participant reiterated that such inequities are exacerbated by a lack of deliberate action towards increasing female representation in leadership:

*There is no deliberate action to have the gender balance for the heads of departments …it’s mainly constituted by men. So, if we have more men head of departments than we do have women, we lose a lot of women to grow up into leadership. So, you find that fewer women qualify for leadership positions than men… So, what we need to do is to encourage greater female representation at the departmental heads. (IDI, Male, #13, MCR)*

Female participants attributed their underrepresentation in leadership and decision-making committees to a range of issues, including incompatibility of women’s gender roles with the nature of science careers, “excuses” given by male leaders that women don't apply for such positions, institutional sexism and bullying, and stereotypical perceptions that women are not strong enough to lead an institution.

Most key informants stated that in their research consortia, positions within the management and decision-making committees were skewed towards more men than women. Notably, the PIs of the three consortia that participated in this study were all male, and only two out of the 11 DELTAS RCS initiatives was led by female PIs. The informants further noted that “the steering committee was mainly made of the PIs of the African partnering institutions and Northern research collaborators, most of whom were male” (KII, Male, #15). The reason provided for this skewed gender representation was that “the main consideration for PIs and co-PIs was based on their expertise as opposed to gender” (KII, Female, #02), as well as historic imbalances. They also noted that funding agencies play a role in perpetuating gender inequities at the leadership level, since they rarely appoint female leaders for executive and management positions; for example, noting that Wellcome Trust-funded research institutes in Africa are headed mainly by men.

## Discussion

This study provides insights into the ways in which institutional-level drivers and processes around access to resources, as well as formal and informal rules manifested through policies and everyday practices and culture at the workplace, intersect with macro-level systems of power to produce gender inequities in the scientific career progression of researchers. We have analysed how the challenges of limited career progression opportunities and research funding uncertainties are shaped by oppressive macro-level forces of power in the wider national and global context, making the institutional environment highly complex and competitive for researchers. Whilst this was considered a salient issue affecting both female and male researchers, we have identified differential gendered impacts. We also found that female researchers at all career stages work in an unconducive environment characterized by negative stereotypical attitudes towards career women, gender biases, bullying and intimidation, and sexual harassment. Such negative behaviours and practices at the workplace are inherent in formal and informal institutional rules which interact with the culture to deter their career advancement. In addition, we found gender inequities in access to social resources such as psychosocial mentoring and female role models, as well as failure to provide physical resources pertaining to mother- and baby-friendly nursing facilities, which disadvantages female researchers. The inflexibility of work policies and culture, which are engrained within the formal rules and informal arrangements, compounds the latter issue, which not only affects women but also perpetuates gender inequities by failing to support men who would like to shoulder care responsibilities. Indeed, women attributed their underrepresentation in scientific leadership and decision-making roles to these experiences, which they felt disadvantaged them in career progression. Social scientists also felt marginalized and disadvantaged in ways that intersected with gender stereotyping.

Although mentoring the next generation of African scientists is acknowledged as necessary and a key to successful and satisfying careers [21], studies have shown that there is a shortage of formal mentoring programmes [22]. Our findings raise questions regarding the nature of mentoring that is offered within the DELTAS Africa initiative, which although apparently equally accessible to women and men is in fact “gender-blind”, in that it caters less to the needs expressed by women for psychosocial support in what they often experience as a hostile environment. This indicates the need to rethink mentorship schemes by embracing a structured approach which is also cognizant of both the career and psychosocial needs of female and male researchers. It has been argued that women benefit more from having senior male career mentors, as they typically tend to have more power and influence compared to women, thus making them more effective for the career advancement of mentees [23]. On the flip side, there are advantages in pairing women scientists with female mentors to offer psychosocial support, as they better understand the barriers women scientists encounter in their careers, and the relationship is often more relaxed [23]. We therefore contend that female researchers may need two types of mentors to help enhance equitable progression in their careers.

Even though women are encouraged to identify their own informal mentors, studies have found that compared to men, women have fewer contacts outside their own institutions who could serve in such roles [24]. Indeed, with fewer women in senior scientific and leadership positions, other studies have found that male researchers are more likely than females to have role models and career and psychosocial mentors who are able and willing to promote their career interests [9, 25, 26]. Relatively few examples of women scientific leaders exist, and even fewer who have managed to effectively balance work and family demands, leading to a lack of female role models in science who can exemplify such balance for women seeking successful careers in this field [27]. In line with other studies, the overall picture is of a prevailing scientific culture that provides inadequate direction and psychosocial mentoring for women, eroding their self-confidence, especially for junior researchers, who feel that they cannot afford to make it to senior scientific and leadership positions [4, 28, 29]. Nonetheless, given that many female researchers experience difficulties balancing their careers with family responsibilities, a huge step in promoting societal and structural change would be to encourage men’s involvement in jointly carrying out domestic responsibilities [21].

Our findings align with other studies in SSA [9, 26, 30–32], which have shown that the inflexibility of formal rules around work policies and culture, and a lack of resource allocation for female researchers with nursing needs, disadvantages women with reproductive responsibilities. Vilnius argues that combining family and career is viewed as a “private affair” for women, resulting in a lack of family-favourable environments in scientific institutions [33]. This implies a need to develop and foster an inclusive conducive institutional work environment that is sensitive to gender and diversity needs through the formulation of clear policies and practices, and proper implementation. For instance, creating work models that support women and men with family responsibilities through provision of lactation areas and on-site childcare centres would enable them to balance their careers, family, and personal well-being, thus overcoming barriers to equitable progression.

Our findings that unfriendly work environments characterized by a spectrum of behaviours and practices shaped by gender dynamics at the (meso-)institutional level, such as bullying and discrimination, sexual harassment, gender stereotypes and biases, and inflexible working hours, disadvantage women align with other SSA literature [8, 9, 34–37]. Women’s narratives in our study concur with the work of other scholars who contend that not only is sexual harassment a recurrent problem for women in research institutions in Africa, but bringing attention to it is still perceived as a dangerous act for women, who may therefore opt not to report it [8, 24, 29]. Such women suffer because of a lack of a safe and unbiased reporting system for seeking help, as well as fear of negative repercussions, jeopardizing their academic standing, and fear of not being believed [38]. This may result in poor mental health [39, 40], as well as discouraging women from career progression. Notably, the fact that men did not report experiencing bullying and intimidation may indicate that perhaps it was harder for them to speak about it, or rather that they had a different understanding of what bullying and intimidating behaviours entail.

We found that women were discouraged from becoming pregnant within the life cycle of a funded project, which constitutes both direct and indirect gender discrimination, in that individual women perceived having to choose between childbearing and a scientific career, and gender bias against female candidates was also reinforced. Our previous paper from this study found that women’s career progression opportunities were acutely influenced by simultaneous requirements to establish scientific research careers and the peak of childbearing and rearing responsibilities [10]. The attitudes of decision-makers, the majority of whom are men, who view child-rearing and research as inherently incompatible contribute to this disadvantage [28]. Other studies have found that such practices are more common in environments where women are underrepresented in positions of power and authority, limiting the promotion of gender-responsive policies that could improve the institutional culture [9, 39, 40]. This implies that institutions should work towards better representation of women in leadership roles.

An intersectional analysis enabled us to provide new insights into how the disciplinary dominance of biomedicine in global health research acts as another axis of power influencing individual researchers. This creates a clustering of disadvantage, as women tend to be more represented in social sciences, which is gendered female and stereotyped as less valuable than the biomedical sciences. Indeed, the relatively limited funding opportunities for social scientists interacts with the gendering of the discipline to entrench disadvantage particularly for female researchers [24, 41].

Our findings show that the dearth of career progression opportunities and research funding uncertainties in SSA are shaped by macro-level structural power relations which intersect with formal and informal institutional rules to create differential outcomes along several intersecting power axes. We have argued that the macro-level forces of neocolonial relationships in funding structures exacerbate the racism in grant allocation as perceived by African scientists. Others have similarly posited that the challenges around funding structures are external to Africa, and are engrained in legacies of colonialism that continue to favour Northern-based researchers as parachute researchers [42]. This problem is exacerbated by requiring grant applicants to hold a faculty position, without acknowledging the biases based on ageism, favouritism, and nepotism in providing tenure that are common in higher education institutions in Africa [9, 30, 43]. The criterion favours PIs from Northern academic institutions, who hold permanent faculty positions, and often contract African researchers to conduct research on a short-term basis, continuing extractive approaches that do not build African institutions.

Indeed, other studies have highlighted the existence of un/conscious gender bias during the grant review process, demonstrating that male applicants tend to have higher success rates than their female counterparts [44, 45]. In addition, such gender biases in funding allocation are more likely influenced by implicit or explicit bias on the part of reviewers, and systemic bias in grant programme designs and academic systems [45]. Reviewer biases may be mitigated through anonymized or double-blind review processes, as well as review training [44–46].

Recent government cuts in overseas development assistance research in the United Kingdom serve as a good example of neocolonialism by illustrating the lack of control that LMIC research partners have in such situations [47]. The effects of such cuts could see employment contracts of research staff terminated or reduced, with direct economic and social impacts which may lead to attrition in the research career sector [47]. Overall, this finding has research and practice implications for the research community and funding agencies who need to promote equity in research funding criteria as well as confront structural racism in grant allocation. For instance, funders may need to challenge the prevailing perception that one must collaborate with a renowned white PI to obtain funding, when communicating about calls for grant applications.

Dependence on an inequitable northern grant funding system is entrenched by macro-level forces of the political economy characterized by limited investment in research by most African national governments. Despite the fact that most academic researchers working at African universities have a joint mandate to teach and perform research, for many of them, the boundaries between these two fundamental responsibilities are fuzzy [48]. With many African governments operating at huge budget deficits, there is little money allocated for research to faculties in public universities, which are most seriously affected by limited research career progression opportunities [49]. The competition for limited opportunities exerts significant pressure on junior researchers, which interacts with institutional and societal power relations to exacerbate inequities. It is evident from our findings that the psychological and economic insecurity of short-term employment contracts creates the sense of a “scary” profession for both female and male early-career researchers [4]. However, the impacts are gendered, with regard to both the responsibility for nuclear as well as extended families assigned to men (and single female parents) and the gendered norms and expectations of female social interaction, which favour collaboration over the “rigid model of hyper-competition” that characterizes the “brutally competitive grant culture of scientific research” [40]. This situation is unlikely to be significantly relieved without expanding the number of sustainable scientific positions for junior and early-career researchers in SSA. Indeed, failure to address this problem of limited career advancement opportunities can lead to “brain-drain” among the newly-minted African scientific health research workforce [42]. The relatively recent expansion of so-called soft scientific research funding to African institutions and the concomitant increase in HRCS funding appear to have outpaced institutional career progression structures, placing particular pressure on less established researchers.

Notably, the DELTAS Africa initiative through AESA recognizes this challenge, and is lobbying with African governments to create viable career pathways for research in universities and to invest more of their gross domestic product in research to reduce the reliance on external funding [50]. In this respect, we also opine that inasmuch as such initiatives continue to prioritize the recruitment and training of individuals, they should also consider whether national institutional structures are adequate and willing to support the career progression of the trained research fellows. Moreover, HRCS initiatives need to consider future career systems that are multidimensional, and which challenge the engrained classic linear pipeline model of career progression [51], as a way of recognizing contextual realities in SSA. Thus there is a need to encourage and support researchers to develop innovative approaches to careers in and out of academia [52]. They may also need to consider a shift away from individual and institutional capacity-strengthening towards creating more enabling institutional environments.

Overall, this study has enabled us to show the relevance of the conceptual framework posited based on a review of existing literature [9], as clearly supported by the current findings. It has contributed new insights into how macro-level systems of oppression shape access to resources, which interacts with formal and informal rules and policies to produce and reproduce gender inequities in the scientific career progression of researchers as a result of social power relations. The remaining constituents of the framework have been explored elsewhere [10]. Notably, in the current study, participants did not refer to gender inequities with resource allocation around office space, research facilities, or equipment, as previously reported in other SSA studies [9, 43, 53]. Perhaps this finding can be explained by the fact that participants were part of an HRCS initiative that necessitated provision of such resources for the fellows.

### Study limitations

Findings from this study should be considered in light of the following limitations. First, while the integrated conceptual framework highlights the intersection of gender with multiple aspects of identity such as language and physical disability, insights about language minorities have been presented in a different paper [10]. However, we were not able to identify researchers who identified as disabled within the sampled consortia and the overall DELTAS Africa initiative. Efforts to identify and recruit such individuals from the wider host and participating institutions in selected consortia were prevented by the need for country-level ethical clearance for each institution. This was not possible within the time constraints of the study. In addition, this meant that we could not embark on document review of the nature and kind of operational institutional-level policies and procedures and their implementation. Second, whilst we acknowledge important developments in gender theory and inclusiveness with regard to problematizing the male/female binary and recognizing lesbian, gay, bisexual, transgender, and queer (LGBTQ+) identities, we did not explore these potential dimensions of participant identities in our study. We did not collect data pertaining to participants’ sexual orientation, due to concerns that in the current discursive context of SSA in which LGBTQ+ orientations are stigmatized, this would likely make participants feel uncomfortable. With regard to gender identity, we asked participants to self-identify their gender; only two gender categories were identified by participants—women and men. This is perhaps unsurprising in the current African social context within which public disclosure of nonbinary or transgender identifies is not encouraged. We are not aware of any individual in the DELTAS Africa initiative who identified as nonbinary or transgender, and the DELTAS reporting system does not offer an opportunity to make such a statement. We acknowledge these gaps in our data as a limitation of the research. Third, participant concerns about anonymity and confidentiality prevented the presentation of nuanced comparisons about their affiliated institutions and consortia. Lastly, we acknowledge the underrepresentation of female PDFs in our sample. This was not by study design: despite significant follow-up efforts, we experienced lower take-up of interview offers by female PDFs. Most of them mentioned that they were either on travel or were busy with field and lab work activities; this may reflect the particularly heavy time constraints experienced by female researchers as discussed in our previous paper based on the same study [10].

Despite these limitations, the findings from this study serve as an avenue for understanding the institutional drivers of inequities, which provides DELTAS Africa consortia and similar HRCS initiatives information on the varied intersectional gendered challenges faced by researchers in their pursuit of a scientific career path within their institutional work environments. Detailed participant recommendations and suggestions on how to address such issues will be presented in a separate paper.

## Conclusions

This study offers an in-depth analysis of the institutional-level drivers and processes that produce gender inequities, by illuminating how social and structural power relations shape the scientific career progression of researchers who are beneficiaries of an HRCS initiative in SSA. Specifically, the intersectional approach to gender analysis elucidated how highly competitive and insecure institutional environments are shaped by macro-level forces at national and global levels. Women’s and men’s differential experiences of this environment are further shaped by institutional power relations, policies, practices, and culture that influence inequities in career progression of female and male researchers. Therefore, understanding and addressing the social power relations within both meso-level institutional environments and macro-level national and global funding policies is necessary to promote equitable career progression opportunities. HRCS funding initiatives need to pay attention to improving institutional work cultures, practices, and policies, as well as contributing to a more conducive sectoral environment for scientific careers through both advocacy and addressing internal systemic biases.

## Data Availability

Individual privacy could be compromised if data were made publicly available. For this reason, data cannot be shared.

## Abbreviations

AESA: Alliance for Accelerating Excellence in Science in Africa
ARIs: African Research Institutions
DELTAS Africa: Developing Excellence in Leadership, Training and Science in Africa
DELTAS ARC: DELTAS Africa Research Consortia
HEIs: Higher Education Institutions
HRCS: Health research capacity-strengthening
IDI: In-depth interview
KII: Key informant interview
LGBTQ+: Lesbian, gay, bisexual, transgender, and queer
LMIC: Low- and middle-income countries
MCR: Mid-career research scientist
MSc: Masters
PDF: Postdoctoral research fellow
PhD: Doctoral researcher
PI: Principal investigator
SSA: Sub-Saharan Africa

## Footnotes

1 https://www.aasciences.africa/sites/default/files/Publications/DELTAS%20Africa%20factsheet_2018_0.pdf site accessed on 6 January 2020.
